# First identification and molecular characterization of protozoan parasites associated with abortion in ruminants from South Sinai Governorate, Egypt

**DOI:** 10.1007/s11259-025-10698-9

**Published:** 2025-03-19

**Authors:** Safaa Mohamed Barghash, Al-Shaimaa Mohsen Sadek

**Affiliations:** 1 Parasitology Unit, Animal and Poultry Health Department, Animal Production and Poultry Division, Desert Research Center, El-Naam, Cairo, Egypt; 2 Parasitology, Zoology and Entomology Department, Faculty of Science, Al-Azhar University, Nasr City, Cairo Egypt

**Keywords:** Abortion, *Toxoplasma gondii*, *Enterocytozoon bieneusi*, *Neospora caninum*, *Sarcocystis* spp., *Microsporidium* spp.

## Abstract

This study presents the first molecular detection and characterization of protozoa associated with livestock abortion, including *Toxoplasma gondii*, *Neospora caninum*, *Sarcocystis* spp., and *Microsporidia* spp., in the South Sinai Governorate, Egypt. A total of 226 blood samples were collected from cattle, goats, and sheep with a history of abortion. Molecular analysis was conducted using PCR and sequencing of the internal transcribed spacer (ITS) region of the rRNA gene. The results revealed the prevalence of *T. gondii* in cattle at 12.33% (9/73), while goats and sheep tested negative. In contrast, 14.92% (10/67) of goat samples tested positive for *Microsporidia* spp., with no cases detected in cattle or sheep. No infections with *Sarcocystis* spp. or *N. caninum* were identified in any tested animals. Across all examined livestock, the overall prevalence of *T. gondii* was 3.98% (9/226), while *Microsporidium* spp. exhibited a slightly higher prevalence at 4.42% (10/226). Molecular sequencing identified three novel *T. gondii* strains in cattle recorded in the GenBank database under accession numbers MZ197902, MZ197903, and MZ197904, showing 99.9% genetic similarity to reference strains. Additionally, three novel strains of *Enterocytozoon bieneusi* (MZ197781, MZ197782, and MZ197783) were detected in goats. Phylogenetic analysis indicated a close evolutionary relationship between these newly identified *E. bieneusi* strains and known genotypes, suggesting a high degree of genetic conservation. These findings underscore the ongoing risks of *T. gondii* and *E. bieneusi* to livestock and public health. Continued surveillance is necessary to assess the zoonotic potential of these novel strains to aid in effective management strategies.

**Supplementary Information:**

The online version contains supplementary material available at 10.1007/s11259-025-10698-9.

## Introduction

Infectious abortion is a significant cause of reproductive failure and economic losses in animals, often linked to bacterial infections such as *Mycoplasma* spp., *Brucella* spp., and *Chlamydia* spp. in Egypt and worldwide (Wassif et al. 2017; Mahmoud et al. 2019; Zhang et al. 2020; Allam et al. 2024; Mahmoud et al. 2024; Shafiei et al. 2024). Protozoan parasites, including *Toxoplasma gondii*, *Neospora caninum*, *Sarcocystis* spp., and *Microsporidia* spp., also cause reproductive problems and abortions in livestock, leading to significant economic losses globally. These pathogens often transmitted under favorable conditions, impact cattle, camels, goats, and sheep, resulting in abortions, stillbirths, and weak offspring (Castro-Forero et al. 2022; Nayeri et al. 2021; Khattab et al. 2022; Barghash 2022a; Mohammed et al. 2023).

*T. gondii* is highly prevalent worldwide, causing fetal death in early pregnancies and weak neonates in later stages. Its zoonotic transmission, primarily through undercooked meat or contaminated environments, poses significant risks to pregnant women and immunocompromised individuals (Nayeri et al. 2021). *N. caninum* is a pathogen that spreads through oocysts in the feces of definitive hosts, such as canines or wild canids, to intermediate hosts like cattle, where tachyzoites differentiate into bradyzoites and form cysts in the host’s muscle and tissue. Similar to *Sarcocystis* spp., the definitive host completes the life cycle by ingesting contaminated muscle tissues (Dong et al. 2018). *N. caninum* can also be transmitted congenitally through transplacental transfer and other pathways (Dubey et al. 2007).

*Microsporidia* spp., particularly *Enterocytozoon bieneusi*, are emerging zoonotic parasites that cause gastrointestinal and reproductive disorders in animals and diarrhea in immunocompromised humans (Han et al. 2021). Animals can readily acquire these protozoan diseases depending on the environmental load of infective spores (Dubey et al. 2007).

South Sinai Governorate, a vital and economically significant region characterized by arid conditions and close human-livestock interactions, faces increased zoonotic risks (Abou-El-Naga 2015; Helmy et al. 2017). The ecological setting, including limited water resources, high livestock density, and vector abundance create an environment conducive to transmitting of protozoan pathogens. Despite their importance, limited data are available on the prevalence and genetic diversity of these pathogens in Egypt, particularly in South Sinai (Barghash 2022a, b, c). Factors such as the survival time and infectivity of oocysts, host behavior, and population density significantly influence the risk of acquiring toxoplasmosis (Meerburg and Kijlstra 2009).

Recent advancements in molecular diagnostics, including PCR and ITS sequencing, enable accurate detection and genotyping of protozoan parasites. These techniques facilitate the identification of novel strains and provide valuable insights into their genetic diversity, which are critical for improving disease control and prevention (Taghipour et al. 2021).

This study aims to investigate the molecular detection and characterization of protozoan parasites associated with livestock abortion in South Sinai. By addressing this knowledge gap, the study provides crucial insights into the epidemiology of protozoan infections in the region, contributing to better disease management and public health strategies.

## Materials and methods

### Study area

**Fig. 1:**
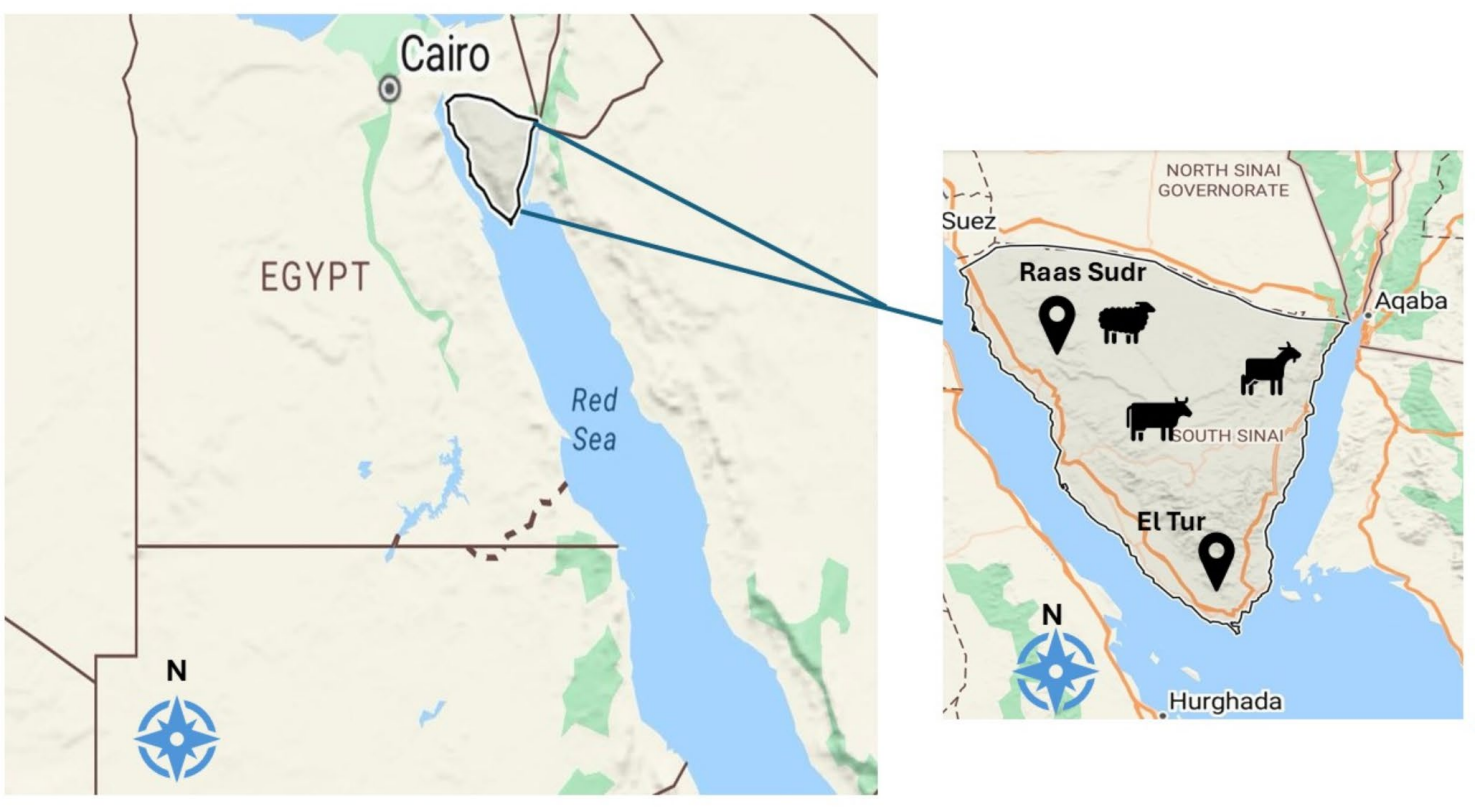
Google map showing the sampling regions and species

The study was conducted in South Sinai Governorate (Ras Sudr and El Tur cities) from April 2020 to September 2021. South Sinai, located at approximately 28.5390° N latitude and 33.9538° E longitude with total area of 31, 272 km2. It experiences an arid desert climate characterized by very low annual rainfall and extreme temperature fluctuations (Fig. 1).

### Animals and sample collection

The protozoan causative agents of abortion were investigated in 226 blood samples collected from female livestock, including 73 cattle, 67 goats, and 86 sheep, from 13 large farms in El Tur and Ras-Sudr. Farms were selected based on a documented history of abortion cases within the past year. The inclusion criteria required farms to raise cattle, goats, or sheep and provide access to relevant records and livestock. Farms with no history of abortion or those unwilling to participate were excluded.

Sampling prioritized animals with a recent history of abortion, as they were considered at higher risk for protozoan infections. While the sample distribution was not strictly proportional to the total livestock population, it ensured adequate species representation. Factors such as farm accessibility, owner consent, and logistical feasibility also influenced sample selection.

The selected farms in South Sinai varied in biosecurity, livestock density) with livestock numbers ranging from 50 to 70 animals per farm), and proximity to wildlife, with most using open grazing systems and limited biosecurity. Feeding modes included a combination of grazing and supplementary feed, depending on the season and availability of forage. Most farms followed regular deworming programs, with deworming occurring biannually. Blood samples were collected from all species’ jugular veins using an appropriate gauge needle and sterile vacutainer tubes containing EDTA as an anticoagulant. The collecting site was cleansed with 70% alcohol before venipuncture. Each animal provided around 5–10 mL of blood, and pressure was given to the puncture site after collection to avoid bleeding (Constable et al. 2016). The samples were immediately placed on ice and transported to the laboratory, where they were kept at 4 °C until further processing.

### Statistical analysis

Chi-square tests were used to evaluate the association between animal species (cattle, goats, and sheep) and the presence of *T. gondii* and *Microsporidia* spp. Contingency tables were created, and expected frequencies were calculated. Significance was determined at α = 0.05 with 2 degrees of freedom (df), rejecting the null hypothesis if the chi-square statistic exceeded 5.99 or if *p* < 0.05.

### DNA extraction and oligonucleotide primers

DNA extraction from samples was performed using the QIAamp DNA Mini Kit (Qiagen, Germany) with slight modifications to the manufacturer’s recommendations. Briefly, 200 µL of the sample suspension was incubated with 10 µL of proteinase K and 200 µL of lysis buffer at 56 °C for 10 min. Following incubation, 200 µL of 100% ethanol was added to the lysate. The sample was then washed and centrifuged according to the manufacturer’s instructions. Nucleic acids were eluted in 100 µL of elution buffer. Primers were supplied by Metabion (Germany) and are listed in Table 1. They were selected based on their relevance to pathogenicity, ease of amplification, and demonstrated performance in previous studies, ensuring comparability (Khattab et al. 2022; Barghash 2022a).

**Table 1:** Primers sequence of the target genes and their cycling conditions

Parasite *(Target gene)*	primers sequences (Forward and reverse)	Size (bp)	Primary denaturation	Amplification (35 cycles)			Final extension	Ref.
				Secondary denaturation	Annealing	Extension		
Microsporidia *(SSU rRNA)*	GGTTGATTCTGCCTGACG	779	94˚C 5 min.	94˚C 30 s.	55˚C 40 s.	72˚C 45 s.	72˚C 10 min.	Askari et al. 2015
	CTTGCGAGC(G/A)TACTATCC							
*T. gondii* *(P30)*	TTGCCGCGCCCACACTGATG	914	94˚C 5 min.	94˚C 30 s.	65˚C 1 min.	72˚C 1.5 min.	72˚C 10 min.	Jones et al. 2000
	CGCGACACAAGCTGCGATAG							
*Sarcocystis* *(18 S rRNA)*	GCACTTGATGAATTCTGGCA	600	94˚C 5 min.	94 °C 30 s	50 °C 40 s	72 °C 45 s	72 °C 10 min.	Bahari et al. 2014
	CACCACCCATAGAATCAAG							
*N. caninum* *(Nc5)*	CCCAGTGCGTCCAATCCTGTAAC	337	94˚C 5 min.	94 °C 30 s	63 °C , 40 s	72 °C 40 s	72 °C 10 min.	Müller et al. 1996
	CTCGCCAGTCAACCTACGTCTTCT							

### PCR amplification

For uniplex PCR, primers were utilized in a 25-µL reaction containing 12.5 µL of Emerald Amp Max PCR Master Mix (Takara, Japan), 1 µL of each primer of 20 pmol concentrations, 4.5 µL of water, and 6 µL of DNA template. The reaction was performed in an Applied BioSystem 2720 thermal cycler. The products of PCR were separated by electrophoresis on 1.5% agarose gel (AppliChem, Germany, GmbH) in 1x TBE buffer at room temperature using gradients of 5 V/cm. For gel analysis, 20 µL of the uniplex PCR products were loaded in each gel slot. Gene ruler 100 bp ladder (Fermentas, Germany) were used to determine the fragment sizes. The gel was photographed by a gel documentation system (Alpha Innotech, Biometra) and the data was analyzed through computer software.

### Sequencing and phylogenetic analysis

The PCR products were purified using a QIAquick extraction kit (Qiagen Inc., Valencia, CA). They were sequenced using an Applied Biosystems 3130 automated DNA sequencer (ABI, 3130, USA). PCR-positive amplicons were purified and sequenced using the Sanger sequencing method. The obtained sequences were analyzed using BLAST (Basic Local Alignment Search Tool) to identify genetic similarities with reference strains in GenBank. Multiple sequence alignments were conducted using ClustalW, and phylogenetic trees were constructed using the neighbor-joining method with bootstrap support (1000 replications) in MEGA software, according to Tamura et al. (2013).

## Results and discussion

**Fig. 2:**
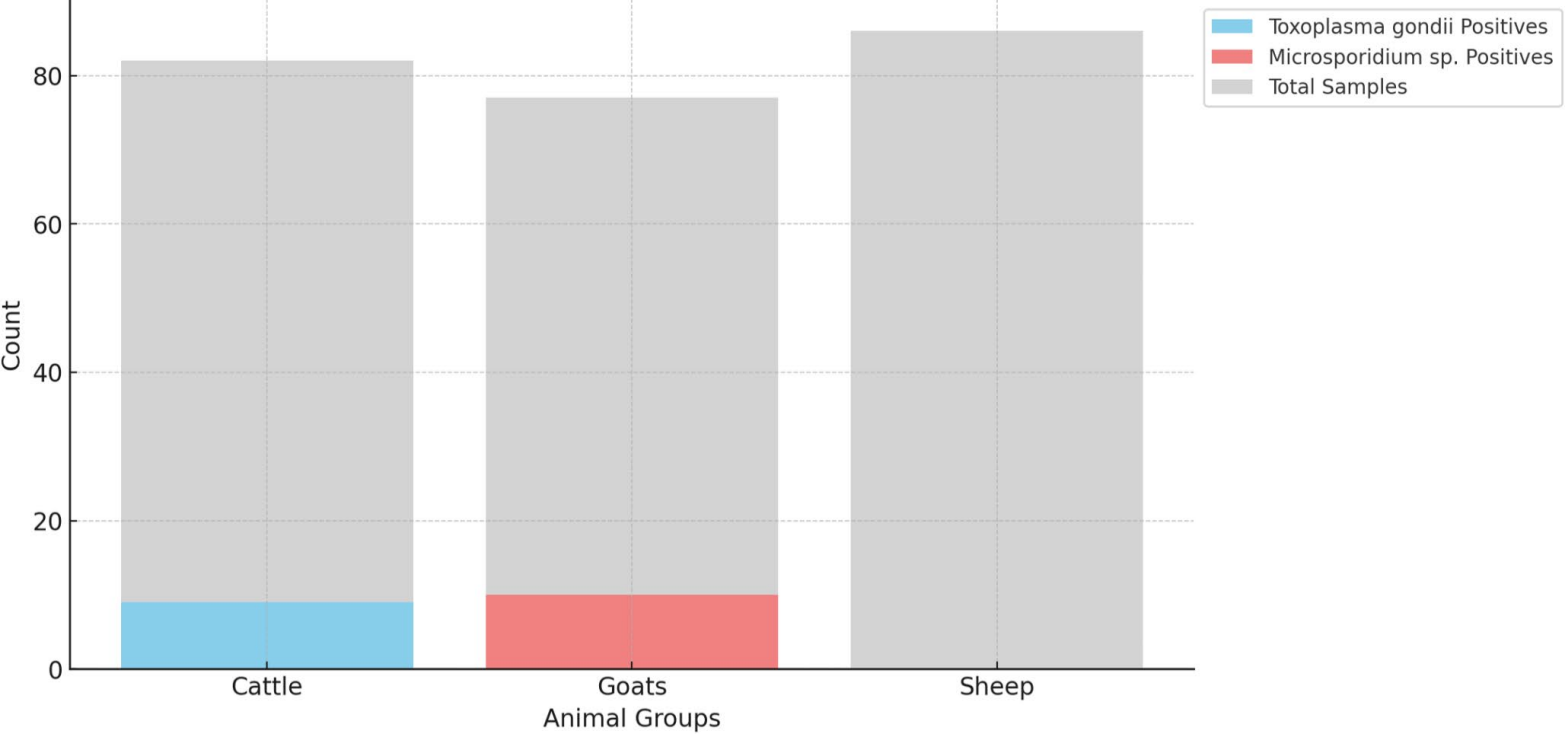
Stacked bar chart representing the total number of samples and positive cases of *Toxoplasma gondii* and *Microsporidia* spp*.* across three livestock groups

This study provides the first molecular detection of protozoa linked to livestock abortions in South Sinai, Egypt, examining 226 samples from cattle, goats, and sheep. The study revealed *T. gondii* in 9/73 (12.33%) of cattle and *Microsporidia* spp. in 10/67 (14.93%) of goats, whereas sheep tested negative for both. Overall infection rates for *T. gondii* were 3.98% (9/226) and 4.42% (10/226) for *Microsporidia* spp (Fig. 2), with no cases of *N. caninum* or *Sarcocystis* spp. (0.0%). These findings align with Khattab et al. (2022), who reported a similar *T. gondii* prevalence in Egyptian cattle (13.46%), though small ruminants exhibited higher rates (43.75% in sheep and 27.93% in goats) and camels recorded a prevalence of 64.51% in northwest Egypt. The chi-square analysis revealed significant associations between the type of animal (cattle, goats, sheep) and the presence of the abortive agents *T. gondii* (χ² = 19.54, df = 2, *p* < 0.05) and *Microsporidia* spp (χ² = 24.93, df = 2, *p* < 0.05). These findings suggest that the prevalence of these pathogens varies significantly among the different animal species studied. They employed the B1 gene to produce shorter fragments of 193 bp and 97 bp for the outer and inner regions, followed by the P30 gene, which yielded a single fragment at 914 bp, preferred for sequencing. In the present study, PCR amplification of the P30 gene produced a single product of approximately 914 bp, which was used for sequencing.

The prevalence of *Microsporidia* spp. in goats was higher than reported by Oladele et al. (2023) but lower than the findings of Al-Herrawy (2016), who documented 17.0% prevalence in animal fecal samples. Intestinal Microsporidium infection rates varied across species, with higher prevalence in dogs (33.3%) and lower in buffaloes (6.9%), suggesting that geographic, climatic, and host-specific factors influence infection rates. The elevated prevalence in goats may be attributed to their open grazing habits and diverse feeding behavior, which increase their exposure to contaminated water sources and environmental *Microsporidia* spores. Additionally, species-specific susceptibility or variations in immune response may also contribute to this trend (Taghipour et al. 2021).

**Fig. 3:**
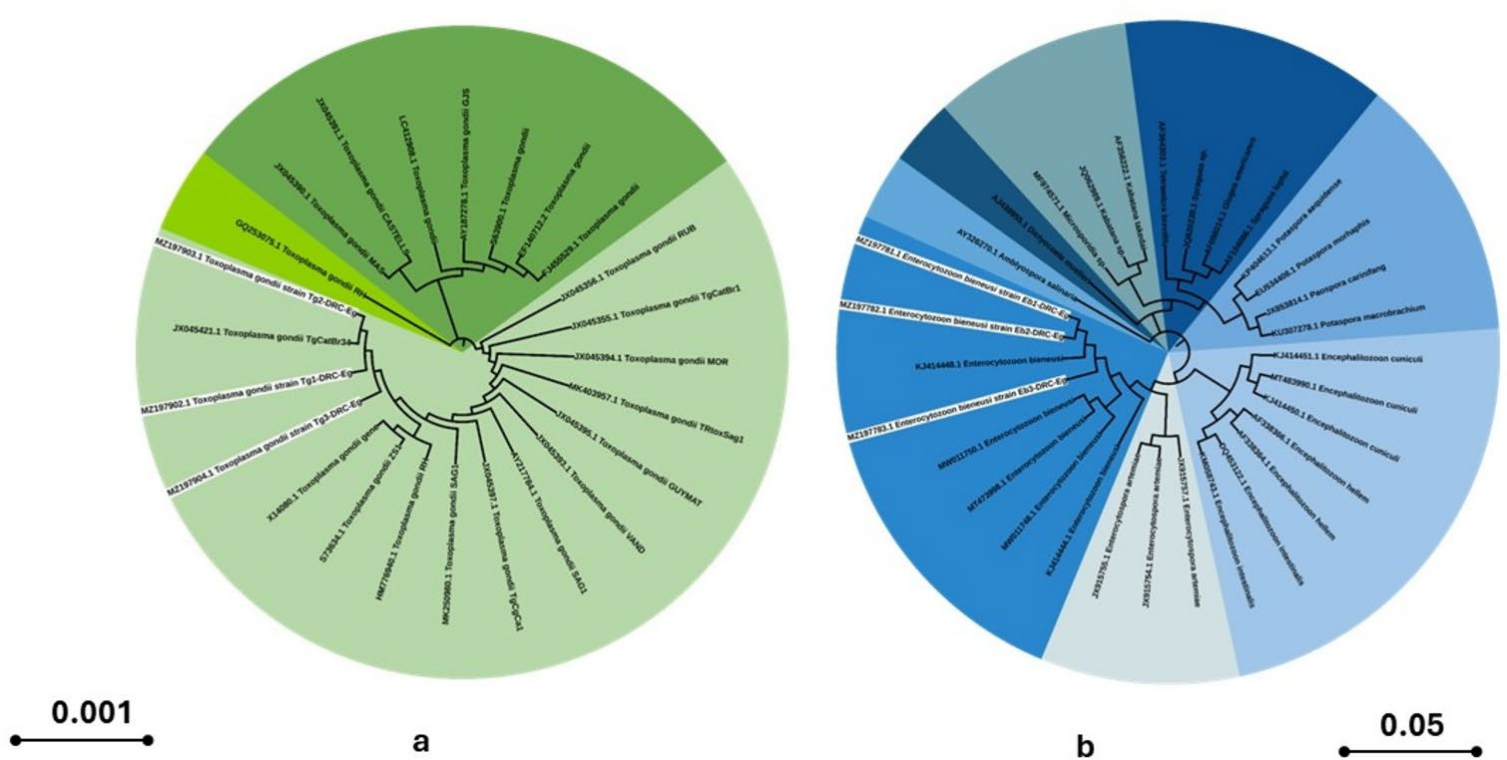
Phylogenetic trees of nucleotide sequences for *Toxoplasmagondii* and *Enterocytozoonbieneusi* isolates recovered from livestock. **a** The P30 rRNA gene region of *Toxoplasmagondii* isolates from aborted cattle, alongside reference sequences from GenBank, shows identities ranging from 100% to 99.8%. **b** The SSU rRNA gene region of *Enterocytozoonbieneusi* isolates from aborted goats, with reference sequences from GenBank, displays identities ranging from 100% to 99.3%. Both trees were constructed using the Tamura 3-parameter model in MEGA software version 6 and visualized with iTOL v6, with our isolates marked on a white background

PCR and gel electrophoresis confirmed these results, with bands at 914 bp for *T. gondii* and 779 bp for *Microsporidia* spp. Phylogenetic analysis revealed novel *T. gondii* strains (MZ197902–MZ197904) with 99.9% identity to global references and new *E. bieneusi* strains (MZ197781–MZ197783), which clustered closely with known strains (Fig. 3a and b). Both species exhibited high genetic homogeneity, with 99.3–100% sequence identity across regions and hosts. *T. gondii* isolates showed perfect identity with strains from North America (JX045421) and other global regions, such as Iran (MK250980) and Malaysia (HM776940), highlighting their genetic conservation crucial for transmission. Similarly, *E. bieneusi* strains demonstrated 99.3–100% identity, with slight regional variations (China: MW011750; Turkey: MT473998) (Table 2). The genetic conservation of the P30 rRNA (*T. gondii*) and SSU rRNA (*E. bieneusi*) genes underscore their evolutionary stability and essential roles in maintaining pathogenicity across diverse hosts and environments. These findings emphasize the zoonotic potential of these pathogens, with significant implications for livestock management and public health. Continued molecular surveillance is vital for understanding their epidemiological dynamics and guiding strategies to mitigate reproductive losses and zoonotic risks (Ahmed et al. 2008; Dubey 2010; Taghipour et al. 2021).

A previous study by Barghash (2022a) investigated whether cattle transported from Delta governorates, where certain *Sarcocystis* infections had been documented, posed risks to newly established farms in South Sinai. The findings revealed *Sarcocystis* infections in 38.9% of cattle but no cases in goats or sheep, indicating a significant etiology. In the current study, the animals examined were from farms across various towns and villages, including cattle settled or brought in for animal husbandry from regions where *Sarcocystis* had not been detected. Additionally, sheep and goats raised locally in South Sinai were examined, and no camels were present on these farms. Thus, it is unsurprising that these animals were free of sarcocystosis and trypanosomiasis, despite *T. evansi* having been previously documented in goats and cattle in other parts of Sinai (Barghash 2022a, b). Variations in farm practices, including management, hygiene, and feeding strategies, may have significantly influenced parasite exposure.

The absence of *Sarcocystis* spp. and *N. caninum* infections in this study may be attributed to the ecological conditions of South Sinai, limited contact with definitive hosts, and effective livestock management practices. Control measures over time may have also reduced the prevalence of *Sarcocystis* spp. Furthermore, diagnostic sensitivity and sampling strategies could influence these results. Climatic conditions play a crucial role in shaping the distribution and diversity of parasitic communities, particularly in arid environments like South Sinai. The harsh desert climate, characterized by extreme temperatures and low rainfall, likely impacts the survival, transmission, and adaptation of protozoan parasites in indigenous livestock (Behnke et al. 2019).

The detection of novel strains of *T. gondii* and *E. bieneusi* has critical implications for public and veterinary health. Their zoonotic potential, coupled with genetic similarities to globally distributed species, underscores the need for continued molecular surveillance to monitor their spread and assess associated risks to humans and animals.

Despite the valuable insights provided by this study, certain limitations should be acknowledged. First, the geographic scope was restricted to South Sinai, which may limit the generalizability of findings to other regions with different ecological and management conditions. Second, while the sample size (226 animals) was sufficient for preliminary epidemiological assessment, a larger and more diverse sampling across different seasons and farm systems could enhance the robustness of the findings. Third, the study relied on PCR amplification of the ITS region, which, while widely used for protozoan detection, may have inherent limitations in specificity and resolution compared to multi-locus genotyping or whole-genome sequencing. Future studies should incorporate larger-scale surveillance, seasonal sampling, and additional genetic markers to further refine our understanding of protozoan epidemiology in livestock.

**Table 2:** The identity percentage between nucleotide sequencing of different regions and hosts and our isolated strains based on P30 rRNA gene for *Toxoplasma gondii* and SSU rRNA for *Microsporidia* spp

Acc. No.	Host	Country	Identity %	Ref.
*T. gondii*				
MZ197902	Cattle	Egypt	100	The present study
MZ197903	Cattle	Egypt	100	The present study
MZ197904	Cattle	Egypt	100	The present study
JX045421	Mice	North America	100	Khan et al. (2009 and 2011)
X14080	Direct Submission to GenBank	------	100	Burg et al. (1988)
S73634	Direct Submission to GenBank	------	100	Chen and Jiang (1994)
HM776940	Direct Submission to GenBank	Malaysia	100	Rahmah and Emelia (2010)
MK250980	Direct Submission to GenBank	Tehran	100	Teimouri et al. (2018)
AY217784	Direct Submission to GenBank	France	99.8	Filisetti et al. (2003)
JX045397	Mice	North America	99.8	Khan et al. (2009 and 2011)
JX045393	Mice	North America	99.8	Khan et al. (2009 and 2011)
JX045395	Mice	North America	99.8	Khan et al. (2009 and 2011)
MK403957	Direct Submission to GenBank	Turkey	99.8	Aksit (2011)
JX045394	Mice	North America	99.8	Khan et al. (2009 and 2011)
JX045355	Mice	North America	99.8	Khan et al. (2009 and 2011)
JX045356	Mice	North America	99.8	Khan et al. (2009 and 2011)
*E. bieneusi*				
MZ197781	Goat	Egypt	100	The present study
MZ197782	Goat	Egypt	100	The present study
MZ197783	Goat	Egypt	100	The present study
KJ414448	Dog	Iran	100	Askari et al. (2015)
MW011750	Canines	China	99.7	Ou et al. (2021)
MT473998	Canines	China	99.3	Ou et al. (2021)
MW011748	Canines	China	99.3	Ou et al. (2021)
KJ414444	Sheep	Iran	99.3	Askari et al. (2015)

## Conclusion

The findings highlight the ongoing risk posed by *T. gondii* and *E. bieneusi* to livestock and human health due to their zoonotic potential. The identification of novel strains underscores the necessity for continued surveillance and molecular characterization of protozoa in livestock populations. These results provide valuable insights into the epidemiology of protozoan infections in South Sinai, with implications for public health and livestock management strategies. Potential transmission routes include contaminated water sources, direct animal contact, and the consumption of undercooked meat or unpasteurized dairy products. To mitigate risks, enhanced biosecurity measures, routine screening programs, improved farm hygiene, and public health awareness campaigns should be prioritized, particularly in high-risk areas. Further research is required to evaluate the zoonotic potential, distribution, and broader epidemiological significance of the newly identified strains. This study represents a significant step toward developing targeted control and prevention strategies at regional and national levels to reduce transmission risks and safeguard both animal and human health.

## Electronic supplementary material

Below is the link to the electronic supplementary material.

Supplementary Material 1

## Data Availability

All data supporting the findings of this study are available within the paper.

## Declarations

### Submission declaration and verification

This manuscript has not been published and is not under consideration for publication elsewhere.
