# Supplementary material for: First identification and molecular characterization of protozoan parasites associated with abortion in ruminants from South Sinai Governorate, Egypt
Source: Vet Res Commun. 2025 Mar 19;49(3):145. doi: 10.1007/s11259-025-10698-9 (PMC11922995; doi:10.1007/s11259-025-10698-9)
Supplement: Supplementary file 1 — Supplementary Material 1 [file 11259_2025_10698_MOESM1_ESM.docx]

**
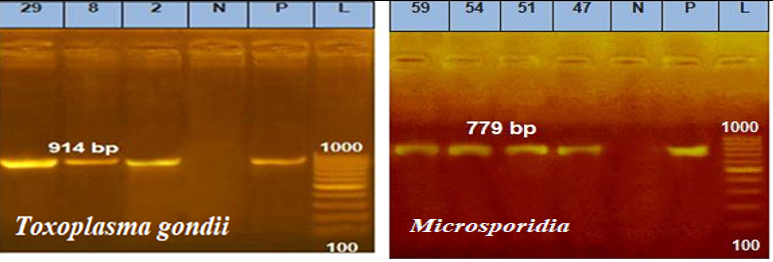
**

**Supplement 1 PCR-based assays targeted the P30 and *SSU rRNA* genes for *Toxoplasma gondii* and Microsporidia, respectively.** *L, L lanes: 100 bp DNA ladder. Lanes P, N, and L represent positive and negative controls, respectively. The other Lanes correspond to positive results for the two parasites in ruminants.*


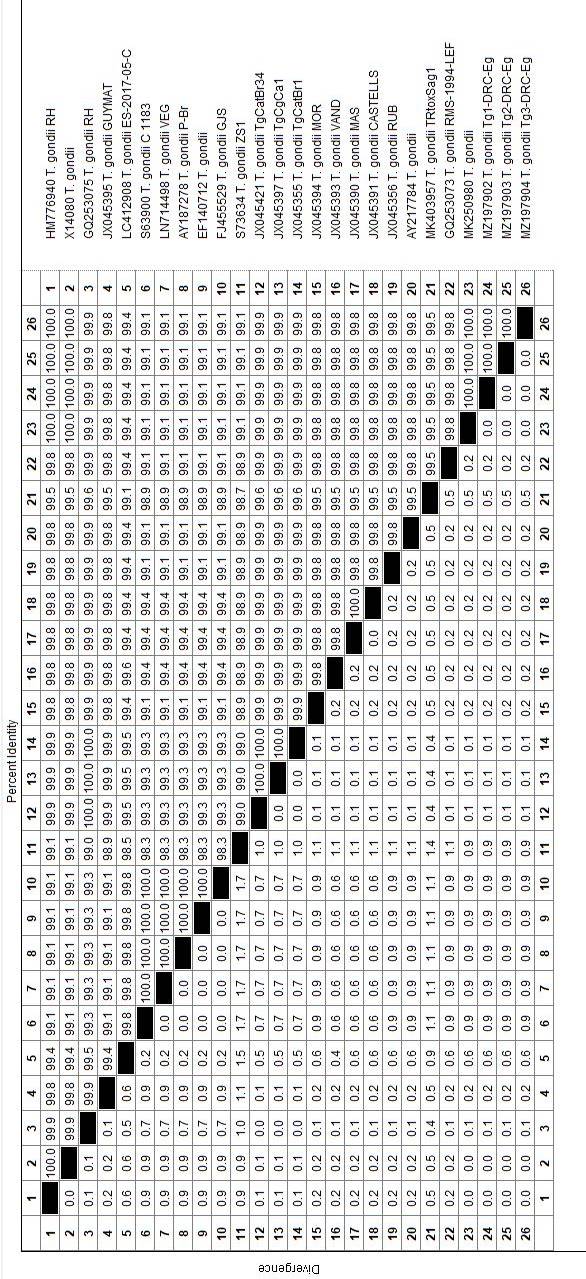
**Supplement 2** The percent of identity for our submitted *Toxoplasma gondii* based on the P30 rRNA gene. *The accession numbers of our submitted isolates are followed by their origin (DRC- Eg).*


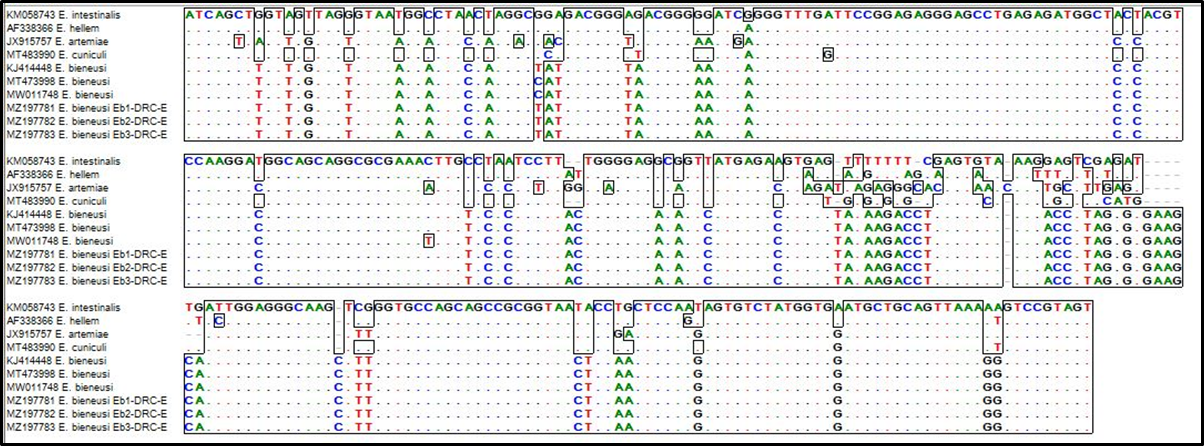


Supplement 3 Total nucleotide sequence differences in the SSU rRNA gene between isolates from the current study and reference sequences of *Enterocytozoon* *bieneusi*. Dots indicate base identical to those in obtained isolates.


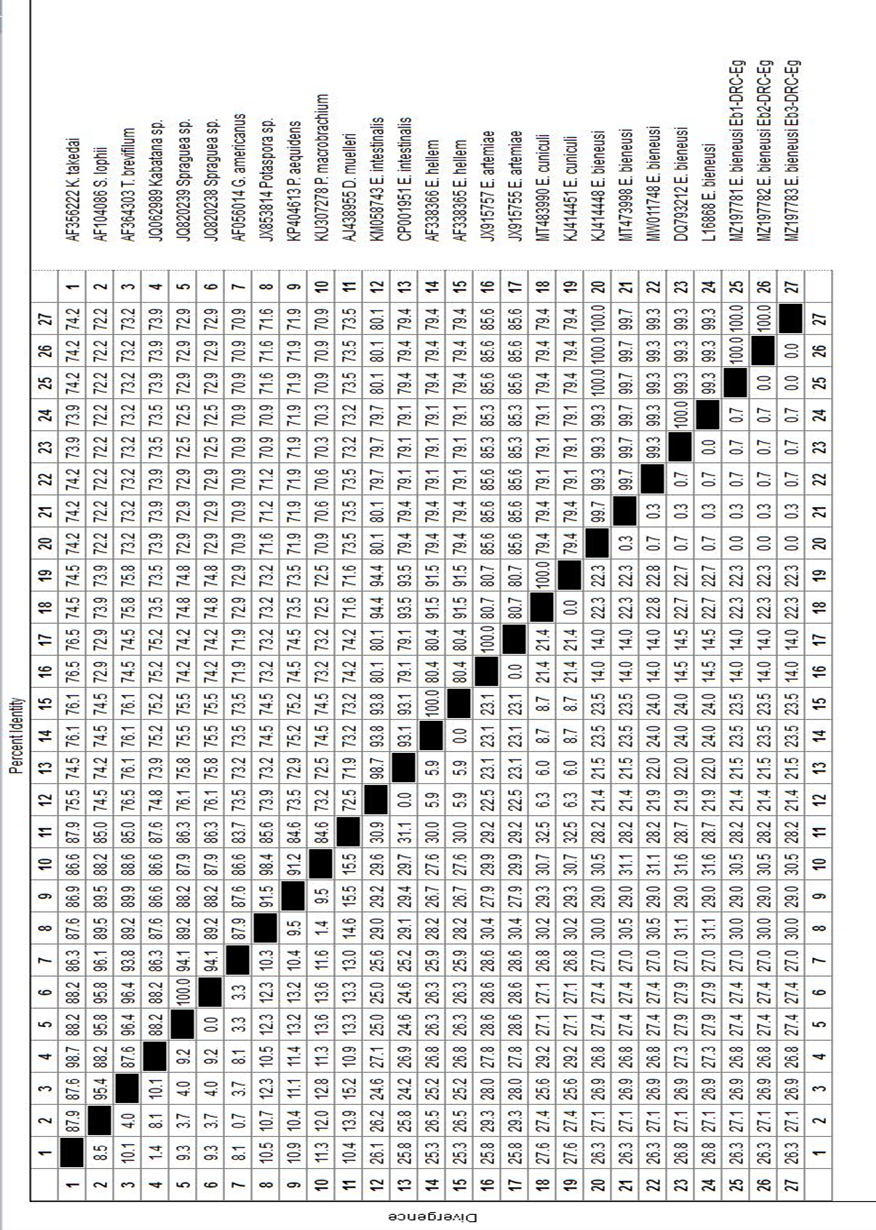


Supplement 4 The percent of identity for our submitted Microsporidia based on the SSU rRNA gene. *The accession numbers of our submitted isolates are followed by their origin (DRC- Eg).*
